# Acoustic Bubbles as Small-Scale Energy Harvesters for Implantable Medical Devices

**DOI:** 10.3390/mi16040362

**Published:** 2025-03-21

**Authors:** Wenbo Li, Anthony Mercader, Sung Kwon Cho

**Affiliations:** Department of Mechanical Engineering and Materials Science, University of Pittsburgh, Pittsburgh, PA 15260, USA; wel102@pitt.edu (W.L.); mercader@illinois.edu (A.M.)

**Keywords:** acoustic resonator, microfluidics, PVDF-TrFE

## Abstract

Piezoelectric acoustic energy harvesting within the human body has traditionally faced challenges due to insufficient energy levels for biomedical applications. Existing acoustic resonators are often much larger in size, making them impractical for microscale applications. This study investigates the use of acoustically oscillated microbubbles as energy-harvesting resonators. A comparative study was conducted to determine the energy harvested by a freestanding diaphragm and a diaphragm coupled with an oscillating microbubble. The experimental results demonstrated that incorporating a microbubble enabled the flexible piezoelectric diaphragm to harvest seven times more energy than the freestanding diaphragm. These findings were further validated using Laser Doppler Vibrometer (LDV) measurements and stress calculations. Additional experiments with a phantom tissue tank confirmed the feasibility of this technology for biomedical applications. The results indicate that acoustically resonating microbubbles are a promising design for microscale acoustic energy-harvesting resonators in implantable biomedical devices.

## 1. Introduction

MEMS (Microelectromechanical System) energy harvesting has long been a focus for biomedical applications, offering the appealing prospect of eliminating the need for internal batteries within the human body. The potential reduction in device size and the elimination of the need for repeated surgical interventions for prolonged device use are significant advantages [1,2,3]. Consequently, researchers have explored various mechanisms to power in vivo devices. Some harness the movement of the human body, converting mechanical energy from bodily movements such as those of the lungs or heart into electrical power [4]. Others rely on external energy sources for transmission, employing methods such as radio frequency (RF) power transmission [5], electromagnetic inductive power transmission [6], and acoustic power transmission. Among these, acoustic wave transmission experiences less attenuation in many cases compared to RF and inductive methods, allowing it to reach deeper within the human body [7]. Additionally, there is a trend of reduced attenuation in human tissue as frequencies decrease, making it advantageous to design energy-harvesting devices within the audible range [8].

Acoustic energy harvesting generally uses a piezoelectric material to transform acoustically excited mechanical movements to electrical energy. Piezoelectric material characterization commonly employs the following equation:

(1) $$D_{i}=d_{ij}\;T_{j}\;+\;\varepsilon \;T_{ik}\;E_{k}.$$

where *i*, *j*, and *k* represent the three spatial dimensions; *D* denotes the electrical displacement; *d* represents the piezoelectric stress coefficient tensor; *T* is the mechanical stress vector; *ε* is the permittivity tensor; and *E* represents the electric field vector [9]. Consequently, increasing the stress vector of the material enhances the power output. Piezoelectric materials typically function under two modes: the *d*_31_ mode, where the applied stress and harvested electric field are perpendicular to each other, and the *d*_33_ mode, where they are parallel. Another crucial parameter to consider is the electromechanical coupling coefficient, *k*, which defines the conversion ratio between mechanical energy and electrical energy. This coefficient acknowledges that a more elastically compliant material will undergo more strain under a load, thereby increasing conversion efficiency compared to a material with the same piezoelectric coefficient, *d* [3].

Various thin-film piezoelectric materials have been developed for energy-harvesting applications, including PZT, BaTiO3, ZnO, PVDF, ALN, etc. Among these, PZT ceramics stands out as one of the most popular choices [10] for their typical advantage of a high piezoelectric coefficient. To enhance this coefficient, microstructures of materials are often refined, or high-piezoelectric constant materials can be integrated into composite materials. However, despite continuous improvements and efforts over the years, the power output of these devices remains relatively small, necessitating ongoing efforts to enhance performance. One common approach involves exploring alternative structural designs beyond conventional diaphragm or membrane configurations. Different designs aim to improve efficiency by either inducing larger strain under a given applied load, such as with the cymbal design [11] and a bi-stable beam [12], or by lowering the resonant frequency to target ambient vibrations, as seen in the arc-based cantilever [13]. These are just a few examples of the diverse design possibilities being explored. It is important to note that while PZT offers favorable properties for energy harvesting, such as a high piezoelectric coefficient, it contains lead and is not biocompatible, making it unsuitable for many implantable devices.

Another commonly used piezoelectric material is PVDF (polyvinylidene fluoride) and its copolymers. Their advantages typically include ease of fabrication, biocompatibility, and flexibility. Although these materials exhibit much lower *d*_33_ and *d*_31_ coefficients compared to ceramics, their greater flexibility results in still lower but more comparable *k*_31_ and *k*_33_ coefficients [14]. Another important consideration is the acoustic impedance mismatch. The acoustic impedance of water is 1.5 MRayl. For human tissue, it widely ranges (e.g., 0.2 MRayl for lungs, ~1.6 MRayl for soft tissue, and 8 MRayl for bone). Piezoelectric polymers offer an acoustic impedance of around 4.5 MRayl. Although this is not an optimal match and can cause wave reflection and reduce power efficiency, polymer materials still outperform ceramics, which typically have acoustic impedances greater than 30 MRayl [10]. In this research, water was used and regarded as an acoustic transmission medium for human tissue due to its similar acoustic impedance level.

Given that the energy level attainable with PVDF polymer is lower compared to PZT ceramic material, utilizing PVDF for low-acoustic power applications can present challenges. In this regard, in macroscale applications, acoustic resonators have been employed to increase the level of harvested acoustic energy. Typical acoustic resonators include tube resonators [15], Helmholtz resonators [16], and, more recently, sonic crystal resonators [17]. Resonator devices are typically significantly larger than the PVDF actuator, usually by one or two orders of magnitude when comparing their largest dimensions and several orders of magnitude larger when comparing their smallest dimensions (e.g., thickness). Consequently, there has been limited exploration in integrating microscale resonators to enhance the energy-harvesting efficiency of PVDF materials. Nevertheless, developing a small-scale resonator capable of amplifying harvested energy while maintaining biocompatibility, flexibility, and ease of fabrication is paramount, particularly for applications in medical implantable devices [18,19].

In the meantime, an acoustic bubble serves as a widely utilized microfluidic tool across various applications. When subjected to an acoustic field, a bubble undergoes volumetric oscillation, surface wave formation on its interface, and/or atomization [20]. This phenomenon has been leveraged for diverse purposes, such as object manipulation [21], rotational control [22], microfluidic channel mixing enhancement [23], liquid pumping [24], drug delivery [25], and propulsion generation [26]. An earlier study has demonstrated the capacity of bubble oscillation to serve as an energy source for harvesting on a cantilever beam [27].

In this article, we endeavored to develop a small-scale acoustic bubble-based resonator operating within a low-frequency range. The harvesting material employed was PVDF-TrFE. This resonator was inspired by our preliminary experimental observations of significant oscillation of polymer bodies when used to encase bubbles, leading to a natural curiosity regarding the potential utilization of this polymer oscillation for energy-harvesting applications. A comparative study of harvesters with and without a bubble resonator was conducted to ascertain whether acoustically resonating bubbles enhance the oscillation amplitude, thereby augmenting the electrical energy. This study confirms the functionality of the acoustic bubble-based resonator.

## 2. Device Design

### 2.1. Design Procedure

To integrate a bubble adjacent to a piezoelectric diaphragm, a pre-designed cavity must be created to securely trap the bubble. Given the frequency-dependent nature of acoustic bubble oscillation, the bubble must be actuated at its resonance frequency to maximize the diaphragm’s performance. Furthermore, as the polarity of electrical charges induced in the piezoelectric material directly depends on whether the applied stress is tension or compression, maintaining the first mode of oscillation is critically important to minimize the cancellation of induced charges and thus to maximize the device performance. To this end, the resonance behavior was initially predicted using analytical models, and the oscillation mode was subsequently validated through preliminary experiments on a PDMS diaphragm observed under a high-speed camera. Finally, a piezoelectric diaphragm was integrated atop the PDMS diaphragm for energy-harvesting studies.

### 2.2. Resonance Prediction for the Device

The individual components of the harvesting system have their own resonances contributing to the overall system performance. Therefore, our goal was to predict and match the resonance frequencies of the associated components, including the diaphragm, bubble, and acoustic input actuator.

We first chose a diaphragm with a diameter of 1 mm and a 20 μm thickness; the first mode in the resonance frequency was 5.7 kHz, which was predicted by an ANSYS^®^ 2022 R1 modal analysis. This frequency was selected for the resonance of the whole device. Consequently, the piezo input actuator was also selected with a resonance peak at 5.7 kHz. Next, the resonance of bubbles trapped in a cylindrical cavity was evaluated. Minnaert derived the acoustic resonance of an air bubble in liquid [28]:

(2) $$f_{0}\;=\;\frac{1}{2\pi }\;\sqrt{\frac{3\gamma P_{0}}{\rho {\left(R_{0}\right)}^{2}}},$$

where *f*_0_ is the resonance frequency of the air bubble, *γ* is the polytropic coefficient, *P*_0_ is the pressure the bubble is under, *ρ* is the density of the surrounding liquid, and *R*_0_ is the radius of the spherical bubble.

The equation was subsequently extended to the Minnaert–Strasberg equation [29] to accommodate various shapes of non-spherical air cavities. Here, the resonance of the cylindrical bubble trapped in the cavity was first estimated using the Minnaert equation, using the equivalent diameter based on the volume. Then, the shape effect was evaluated using a prolate spheroid bubble with an eccentricity, which was previously developed and utilized for predicting the resonance of PDMS diaphragm oscillations [30], showing that the resonance frequencies exhibit minimal dependence on the bubble shape. For example, even when the major semi-axis of the spheroid bubble is twice as large as the minor semi-axis, the change in the resonance frequency is approximately limited to within 2% from the equivalent spherical bubble [29]. Note that the Minnaert equation was originally developed for an air bubble in liquid. However, its application for diaphragm-covered bubbles that do not have any liquid–air interface was demonstrated to be adequate [30].

In the present study, for a cylindrical bubble with a diameter of 1 mm and a depth of 1 mm, the radius of the spherical bubble for the equivalent volume was 572 μm, which predicted a resonance frequency of 5.7 kHz. The half-depth of the cavity approximately corresponds to the major axis of the spheroid shape, while the radius of the cavity represents the minor axis. Given that in our designs investigated in this study, the ratio of the axes in our design varied from 1 to 2, the shape-induced variation in bubble resonance was estimated to be within 0.1 kHz. Therefore, shape influence could be neglected when predicting resonance.

### 2.3. Diaphragm Oscillation Visualization

**Figure 1:**
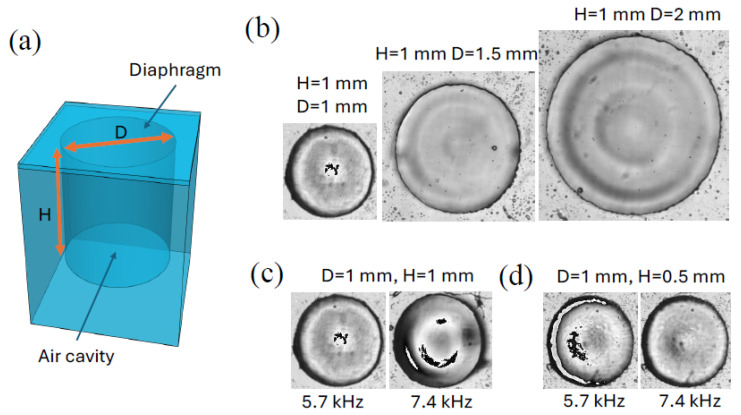
(**a**) Three-dimensional sketch of the tested air cavity covered with a PDMS diaphragm. (**b**) Effect of cavity diameter on oscillation mode (acoustic input: 5.7 kHz, 20 Vpp; video clip: Supplementary Video S1). As the diaphragm diameter grew larger, higher oscillation modes developed on the diaphragm. (**c**) The first mode oscillation occurred at 5.7 kHz for 1 mm diameter and 1 mm depth cavity. (**d**) When the cavity height changed from 1 mm to 0.5 mm, the frequency for the first mode oscillation changed from 5.7 kHz to 7.4 kHz. Supplementary video clip for (**c**,**d**): Supplementary Video S2.

To experimentally validate the aforementioned predictions, the oscillation modes of the PDMS diaphragms were evaluated under the observation of a high-speed camera. In this context, an air cavity with a diaphragm but devoid of a piezoelectric layer was initially fabricated utilizing PDMS. A PDMS diaphragm was spin-coated and subsequently bonded with a PDMS cylindrical cavity, as depicted in Figure 1a. Multiple designs of the cavity were fabricated, varying its diameter and height to investigate their impact on resonant frequency and mode shapes. For all designs, the membrane thickness remained constant at 20 μm. A PDMS diaphragm device was positioned within a water tank (20 cm by 20 cm by 10 cm), to which a piezo actuator for acoustic wave generation was attached. High-speed camera footage was captured to observe the oscillation mode of the PDMS diaphragm as the frequency of the input signal to the piezo actuator was swept.

The results presented in Figure 1b,c demonstrate that the mode number in oscillation increases with frequency. It is challenging to discern the oscillation modes from the static images, although mode shapes were more readily identifiable in the high-speed video clips (Supplementary Videos S1 and S2). To address this limitation, individual frames from the video were extracted and converted into RGB images. Subtraction of corresponding pixel values between frames was subsequently performed to visually illustrate the mode shapes. Notably, the resulting images in Figure 1b,c contain regions of black or white, indicating that the pixel values after subtraction fell outside the defined RGB range. These regions typically corresponded to the peaks and valleys of the oscillation mode shapes of the diaphragm.

Figure 1b (Supplementary Video S1) demonstrates the impact of cavity diameter (1, 1.5, and 2 mm) on the resonance mode when the cavity height is fixed at 1 mm and the applied frequency is fixed at 5.7 kHz. As the cavity diameter increases, the mode (wave) number in the oscillation mode also increases. Figure 1c,d (Supplementary Video S2) illustrate the effect of cavity height (0.5 and 1 mm) on the resonance while the diameter is fixed. Both cases exhibit the first mode, but the resonance frequency shifts from 5.7 kHz to 7.4 kHz when the cavity height changes from 1 mm to 0.5 mm. Based on these findings, the current energy-harvesting device utilizes a cavity with a diameter of 1 mm and a depth of 1 mm.

### 2.4. Fabrication

**Figure 2:**
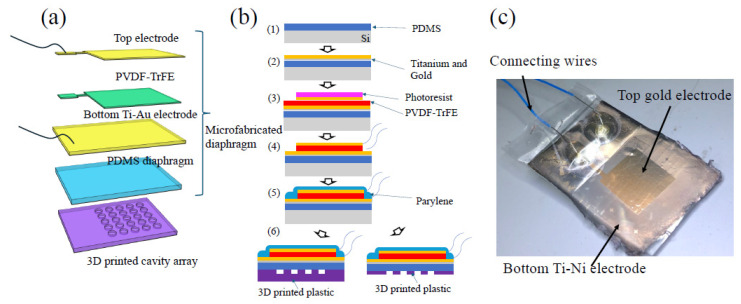
(**a**) Split-up illustration of fabricated device: microfabricated diaphragm and 3D-printed cavity array. (**b**). Step-by-step fabrication process: (1) PDMS spin-coating; (2) O_2_ plasma treatment and bottom-electrode deposition; (3) PVDF-TrFE spin-coating, top-electrode deposition, and photolithography; (4) removing photoresist and excess PVDF-TrFE with O_2_ RIE and attaching lead wires; (5) insulating device with parylene deposition; and (6) detaching from substrate and combining with 3D-printed cavity array for testing. (**c**) Photograph of fabricated device.

The fabrication of the energy-harvesting device was divided into two distinct phases (Figure 2a). The first phase involved the assembly of a piezoelectric layer sandwiched between the top and bottom electrodes on the PDMS diaphragm. The second phase entailed the fabrication of a 3D-printed cavity array substrate. The first phase was executed utilizing conventional microfabrication techniques, while the second phase was accomplished through stereolithography (SLA) 3D printing.

Figure 2b shows the detailed microfabrication steps of the PDMS diaphragm with the piezoelectric layer and electrodes. PDMS (Sylgard 184) was chosen as the diaphragm material due to its low Young’s modulus and ease of fabrication. First, a silicon wafer was treated with silane for the easy detachment of PDMS later. PDMS solution was subsequently poured onto the molds and spun using a spin-coater (Laurell Technologies, North Wales, PA, USA) to achieve a 20 μm diaphragm thickness, which could be detached from the silicon wafer later (Figure 2b(1)). To enhance the adhesion between the PDMS and the bottom gold electrode, the PDMS surface was treated with O_2_ plasma to roughen the surface. Subsequently, a 10 nm adhesion titanium layer was applied before the 30 nm gold layer was deposited for the bottom electrode (Figure 2b(2)). PVDF-TrFE (Solvene 300/P300 from Sigma Aldrich, Burlington, MA, USA) was procured and dissolved in an MEK solution (78-93-3 from Sigma Aldrich) at a 1:10 mass ratio. The solution was then heated at 80 °C and stirred on a hotplate for 30 min to ensure complete dissolution. Subsequently, the solution was spin-coated at 1500 rpm to achieve a uniform thickness of 3 μm. Another layer of 30 nm of gold was then deposited for the top electrode. Photolithography was employed to pattern the top electrode (Figure 2b(3)). However, after etching the metal layer, the residual photoresist on top of the electrode had to be removed. However, since acetone also removed the PVDF-TrFE layer, O_2_ plasma RIE (50 sccm O_2_, 200 mW power) was instead employed to strip both the PVDF-TrFE and the photoresist residue (Figure 2b(4)). To ensure water insulation, the entire device was uniformly vapor-deposited with a 3 μm layer of parylene (Figure 2b(5)).

Following fabrication, the electrodes of the device were connected to output wires using silver paste for electrical connection. Then, electric poling was carried out for the PVDF-TrFE layer to determine its piezoelectric properties. Since piezoelectricity exhibits a Curie temperature of 115 °C, the device was heated on a 120 °C hotplate. Subsequently, a direct current voltage of 180 V was applied across the device electrodes for a duration of 30 min. Subsequently, the heat was gradually removed before the voltage was deactivated. This procedure ensured that the formed crystalline structure remained fixed in a uniform direction.

Finally, the cavity array (5 × 5) part was then fabricated using 3D SLA printing and glass laser cutting. The PDMS diaphragms detached from the silicon wafer were bonded onto the top of the cavity array part (Figure 2b(6)). Figure 2c presents a photo of the fabricated device.

## 3. Results

**Figure 3:**
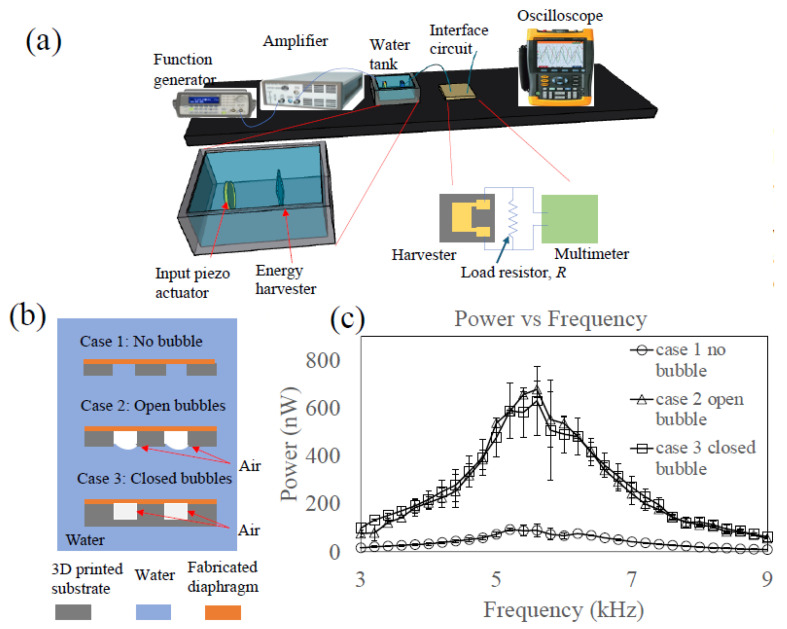
(**a**) Experimental setup. (**b**) Three testing cases: case 1 (no bubble); case 2 (open bubble); and case 3 (closed bubble). (**c**) Harvested power under 20 Vpp voltage: cases 2 and 3 harvested about 7 times more power than case 1.

Figure 3a shows the experimental setup. The fabricated device was immersed in a tank filled with water to simulate a biomedical environment. A piezo bender, coated with parylene for waterproofing, was positioned 5 cm away from the harvester device. The power output of the device was tested using an interface circuit comprising a load resistor of *R* = 80 kΩ, and the voltage output was measured using a reader from National Instrument, USB-6008 with Matlab R2016a. The power, *P*, was then calculated from the measured voltage, *u*, and resistor, *R*:

(3) $$P=\frac{u^{2}}{R}.$$

### 3.1. Power Generation of Three Different Configurations

Three different configurations—(1) case 1: a free-standing diaphragm, (2) case 2: a one-sided open cavity, and (3) case 3: a closed cavity—were evaluated, as shown in Figure 3b. All three configurations used the same structure of the piezo–PDMS diaphragm, but the cavity structures were different. The first configuration had no bubbles trapped in the cavity (1 mm diameter, 200 μm cavity height). To ensure that water completely wetted the cavity, a plasma treatment was applied to the open cavity area to render the cavity surfaces hydrophilic after integrating the PDMS–piezo diaphragm. The second configuration had air bubbles trapped in the cavity (cavity height: 1 mm, diameter: 1 mm). Upon integration with the PDMS–piezo diaphragm, the open cavity surface remained inherently hydrophobic. As soon as the device was immersed in the water, bubbles were trapped in the cavities such that the top sides of the bubbles were in contact with the piezo–PDMS diaphragm while the bottom sides of the bubbles were in direct contact with the water (open bubbles). The contact angle of the water in the cavity was around 100°. The third configuration had the same cavity dimensions as the second case (open bubbles), but the bottom side was closed by a PDMS layer (thickness: 1 mm).

The oscillation mode was once again verified visually with high-speed camera video clips, which showed that all three cases operated under the first mode of oscillation (Supplementary Video S3). Then, the power output of the device was measured with a load resistor of 80 kΩ under a 20 V_pp_ input voltage to the piezo bender. The results (Figure 3c) indicated that the power outputs of the devices with the open bubbles (case 2) and closed bubbles (case 3) had maxima at around 5.7 kHz, nearly identical to each other. Both cases showed a power output approximately seven times higher than that of the free-diaphragm device (case 1). This suggests that the enhanced oscillation of the diaphragm due to bubble resonance enables harvesting of much more energy from acoustic waves.

It is noteworthy that all three cases exhibited similar resonant frequencies. As discussed in the previous section, the diaphragm size, thickness, and bubble size were carefully designed to ensure that their resonance frequencies matched at 5.7 kHz. Notably, deviations from a perfectly spherical bubble shape had a minimal influence on resonance. Similar to the derivation of the Minnaert equation, surface tension effects (including the contact angle) were not considered, regardless of whether a bubble was formed within a 3D-printed material or at a liquid–gas interface. Based on the results for cases 2 and 3, the surface tension (which might include the contact angle) did not seem to affect performance significantly. The volume of the bubble primarily determines the resonance frequency of the structure.

Throughout the testing period, which lasted over one hour, the devices in all three cases remained stable and produced consistent results across multiple trial runs. However, the open bubble in case 2 was expected to gradually dissolve into the liquid phase. Nonetheless, since the bubble size was on the millimeter scale rather than the micrometer scale, dissolution was not significant within the duration of the experimental testing. The results shown in Figure 3c were reproduced across three separate devices, demonstrating the repeatability of the findings.

### 3.2. Oscillation Amplitude and Output Voltage Verification

**Figure 4:**
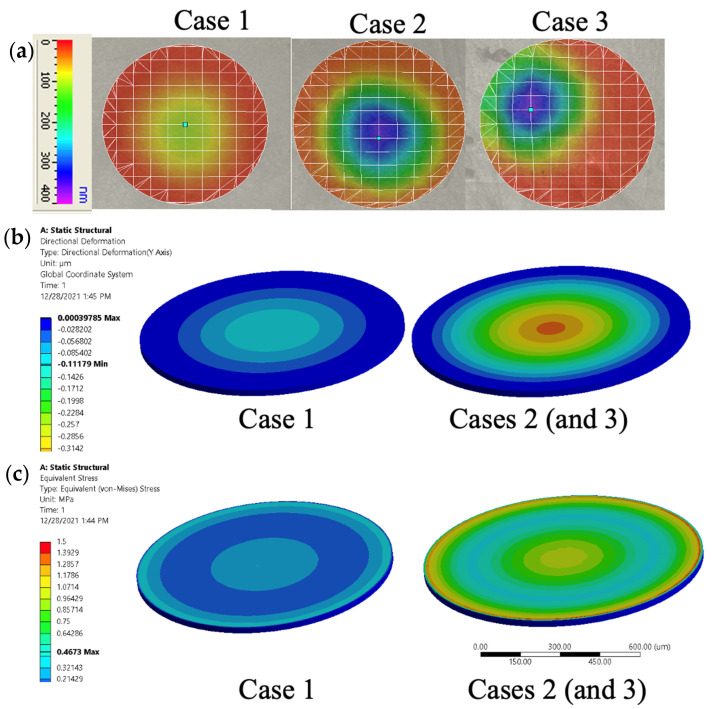
(**a**) LDV measurements of oscillation amplitudes of the diaphragms. The deflection amplitudes of cases 2 and 3 were about 3 times larger than that for case 1. (**b**) Diaphragm deflection determined by Ansys simulations using the maximum deflection from LDV measurements. (**c**) Stress distribution in the diaphragms determined by Ansys simulations.

To verify that the observed power amplification indeed stemmed from the amplification of oscillation amplitude by the trapped bubbles, a Laser Doppler Vibrometer (LDV) was used to measure the oscillation amplitude of the diaphragm, as shown in Figure 4a. For case 1, the amplitude was 111 nm. For cases 2 and 3, the amplitudes were 359 nm and 351 nm, respectively.

The amplitudes measured were used to verify the generated power level using ANSYS simulations. First, the pressure in the cavity was changed in ANSYS simulations until the deformations in the ANSYS simulations were similar to the oscillation amplitudes in the LDV measurements. The stress distributions when the maximum deflections in the ANSYS simulations were equal to the corresponding oscillation amplitudes in the LDV measurements are shown in Figure 4b,c. Since the oscillation amplitudes were measured as being almost identical for cases 2 and 3, the color contours for the deflections and stress distributions obtained by the ANSYS simulations are not distinguishable between cases 2 and 3. Thus, the results for case 2 only are shown in Figure 4b,c, for clarity. For cases 1–3, the simulations proved that the max stresses in the diaphragm were 0.5, 1.49, and 1.47 MPa when the deflections were 111, 359, and 351 nm, respectively, which allowed us to calculate the voltage generated as follows [31]:

(4) $$V=g_{31}\times \sigma_{3}\times t$$

where *V, g*_31_*, σ*_3_, and *t* represent the voltage generated, the piezoelectric voltage constant, the applied mechanical stress, and the thickness of the material, respectively. Given that *g*_31_ = 216 mV∙m/N [31], the calculated voltage outputs were 49, 167, and 164 mV (the expected power outputs were 30, 348, and 334 nW) for the free diaphragm (case 1) and bubble diaphragms (cases 2 and 3), respectively. These predictions were in the same order of magnitude as the experimental results, shown in Figure 3c, confirming that the enhancement of diaphragm oscillation through bubbles in the cavities can result in an enhancement in the harvested energy level. The substantial oscillation of the diaphragm allows the energy-harvesting piezo layer to operate in the *d*_31_ mode.

Note that the oscillation amplitude of the diaphragm does not significantly depend on whether the bubble is open to the water or closed by the PDMS solid wall. According to the Minnaert equation, the resonance frequency is determined by the size of the acoustic bubble and has been used to predict the resonant frequencies of open bubbles [28], partially open bubbles [32], and a solid closed cavity [30]. In addition, the Minnaert–Strasberg equation [29] demonstrated that the influence of bubble shape on the resonance is relatively insignificant. A further study [30] showed that the influence of the surrounding materials is also insignificant. Thus, the similarity in oscillation amplitudes between cases 2 and 3 may be attributed to the dominant influence of air compressibility on the acoustically excited bubble oscillation. Regardless of whether the lower part of the bubble is exposed to the water or closed by the PDMS wall, similar air compressibilities for both cases may lead to comparable effective stiffnesses and thus similar oscillating behaviors.

### 3.3. Impedance Matching and Distance Influence

**Figure 5:**
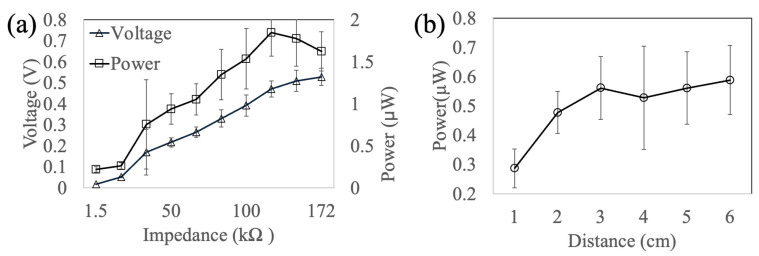
(**a**) Effect of testing circuit impedance (load resistor, R) on harvested voltage and power under 5.7 kHz, 20 V_pp_ input to piezo bender. An optimal impedance is 120 kΩ. (**b**) Effect of distance on harvested power: the harvested power increases as the distance increases from 1 to 3 cm and stays at a similar level when the distance is between 3 and 6 cm (5.7 kHz, 20 Vpp input to the piezo bender).

Impedance matching between the device and the load was evaluated to optimize the power output. When using the same interface circuit but changing the load resistance from 1.5 to 172 kΩ, the system reached the maximum power of 1.8 μW with a 120 kΩ load resistor (Figure 5a).

The distance between the acoustic input piezo actuator and the energy-harvesting device was also investigated. The harvested power vs. the distance is depicted in Figure 5b. When the distance is within 3 cm, the power level increases with the distance. However, between 3 and 6 cm, the distance does not significantly affect the power level.

When the acoustic wavelength is much smaller than the harvesting distance between the emitter and the harvester, it can greatly influence the harvested energy level. However, in the present case, the acoustic wavelength in water at this frequency was around 10 cm, which was half the dimension of the water tank and twice the distance between the acoustic emitter and the harvester. In this scenario, the oscillation of the diaphragm was mainly due to bulk pressure variance rather than a travelling acoustic wave. Therefore, the variance in the power level was most likely caused by the different acoustic power levels in different locations within the water tank due to wave reflection and interference [33]. Possibly, standing waves could affect the acoustic power as well.

### 3.4. Phantom Tissue Simulation

**Figure 6:**
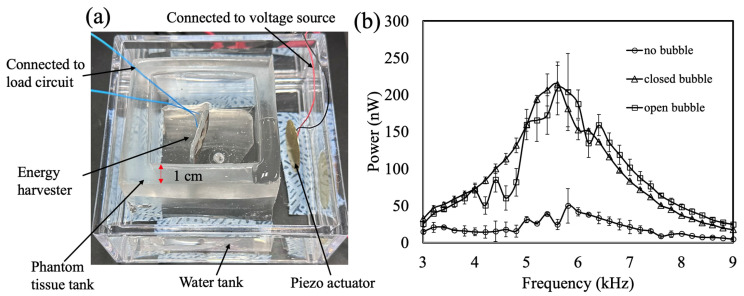
Testing setup of harvesters in phantom tissue tank. (**a**) Experimental setup: The energy harvester was placed in the phantom tissue tank, which had a wall thickness of 1 cm, which was placed inside the water tank. The piezo actuator was placed outside the phantom tissue tank. (**b**) Harvested power when 20 Vpp was applied to the piezo bender. The harvested power levels were significantly higher when the bubbles were installed, although they were lower compared to the results in Figure 3c, due to the attenuation of acoustic waves through the phantom tissue.

To further simulate an in vivo application of this harvester under acoustic attenuation, the device was enclosed by a solid phantom tissue, which was made from PDMS (curing agent ratio: 20:1) to introduce a mismatch in acoustic impedance [34]; the acoustic impedance of this material is around 1.08 MRayl. The PDMS was molded into an open tank shape (6 cm by 6 cm by 10 cm for the outer dimensions, with a thickness of 1 cm) and placed inside the testing water tank (20 cm by 20 cm by 10 cm), separating the harvester and the piezo bender (Figure 6a). The distance between the piezo bender and the harvest device was 5 cm. The input acoustic wave transmitted through the water between the phantom tank and the water tank, the 1 cm thick phantom tissue wall, and the water in the phantom tissue tank to the harvester.

The harvested power from this setup is presented in Figure 6b. As with the previous experiment, the three cases were compared. Despite the decrease by approximately two-thirds in the harvested power due to the phantom tissue compared to the results in Figure 3c, the effect of the bubbles on the harvested power was clearly shown. This result reaffirms the role of acoustic bubbles in enhancing energy-harvesting efficiency. Furthermore, it demonstrates the potential for this effect to be applied in vivo to improve energy harvesting. Less attenuation can potentially be obtained when the acoustic impedance of the phantom tissue matches that of water. Future studies might focus on in vivo evaluations for feasibility at a practical level.

## 4. Discussion

The device presented in this study has the potential to be a cost-effective solution for generating higher power outputs when exposed to acoustic waves. Although microfabrication was used to fabricate the electrodes, it may not be necessary, as neither the electrode dimensions nor the bubble dimensions require microscale fabrication. The materials used in this study, such as the piezoelectric polymer and the PDMS, are readily available and inexpensive, such that the fabricated devices are cost-effective.

Three other studies [15,16,17] were selected for comparison, summarized in Table 1, where the harvested powers and the resonator sizes from these studies were compared with those of the present work. The present harvester and the MEMS harvester [15] showed greater power densities than the other macroscale energy harvesters [16,17]. In particular, the present harvester showed the greatest power density.

Obviously, there is room for improvement of the current design to further increase the harvested energy. Currently, the piezo diaphragm is incorporated only on the front side of the cavities. An additional piezo diaphragm could be added to the backside of the cavities to double the harvested power. Another improvement would be the utilization of higher-order oscillation modes, where a certain portion of the induced electric potential is generally cancelled out when a single piece of electrode is used. To eliminate this cancellation, separated multiple electrodes could be used with polarity regulation in the interface circuit. This method may potentially increase the stress level in the diaphragm and eventually enhance the harvested energy level.

## 5. Conclusions

This paper presents a microacoustic energy harvester utilizing a microbubble resonator, which is much smaller than existing acoustic resonators. PVDF-TrFE is used as the piezo material on a PDMS diaphragm that encapsulates a microbubble amplifying the structural resonance and thus the energy-harvesting efficiency. The geometries of the diaphragm and the bubble were carefully designed to match and maximize the structural resonance of the piezo layer, diaphragm, and bubble. The maximum harvested power was measured at 1.8 μW, which was seven times higher than the case without the bubble. This was due to the fact that the bubble resonator creates much larger displacements and stresses in the diaphragm. The measured power level is in the same order of magnitude as the prediction obtained using the oscillation amplitude of the diaphragms measured by Laser Doppler Vibrometry (LDV) and the maximum stress calculated by ANSYS structural simulations. In addition, a similar device performance was also demonstrated when the acoustic wave was transmitted through a PDMS phantom tissue. The current harvester can be applied in implantable biomedical devices, since acoustic waves of low frequency (<9-kHz) can reach deeper spots in the human body than other types of waves (ultrasound, electromagnetic waves, etc.).

## Figures and Tables

**Table 1:** Comparison of harvested power of the present device with those of other harvesters.

Works	Power Density (μW/cm^2^)	Resonator Size (mm^3^/Single Unit)
MEMS harvester [15]	0.34	1950
Acoustic energy harvesting with Helmholtz resonator and piezoelectric cantilevers [16]	0.02	55,500
Acoustic harvester by piezoelectric curved beams in the cavity of a sonic crystal [17]	9.6 × 10^−5^	384,845
Present harvester	1.85	0.785

## Data Availability

Data will be made available upon reasonable request.

## Supplementary Materials

The following supporting information can be downloaded at https://www.mdpi.com/article/10.3390/mi16040362/s1: Supplementary Videos S1–S3, Video S1, diaphragm response for two diaphragm sizes; Video S2, diaphragm response for different cavity depths; Video S3, oscillation mode verification for fabricated device.
